# Germline mutations in *BRCA1* and *BRCA2* in epithelial ovarian cancer patients in Brazil

**DOI:** 10.1186/s12885-016-2966-x

**Published:** 2016-12-03

**Authors:** Simone Maistro, Natalia Teixeira, Giselly Encinas, Maria Lucia Hirata Katayama, Vivian Dionisio Tavares Niewiadonski, Larissa Garcia Cabral, Roberto Marques Ribeiro, Nelson Gaburo Junior, Ana Carolina Ribeiro Chaves de Gouvêa, Dirce Maria Carraro, Ester Cerdeira Sabino, Maria del Pilar Estevez Diz, Roger Chammas, Geertruida Hendrika de Bock, Maria Aparecida Azevedo Koike Folgueira

**Affiliations:** 1 Departamento de Radiologia e Oncologia, Centro de Investigação Translacional em Oncologia, Instituto do Câncer do Estado de São Paulo, Hospital das Clínicas, Faculdade de Medicina da Universidade de São Paulo, São Paulo, SP Brazil; 2 Faculdade de Medicina da Universidade de São Paulo, São Paulo, SP Brazil; 3 Departamento de Doenças Infecciosas, Instituto de Medicina Tropical, Universidade de São Paulo, São Paulo, Brazil; 4 Departamento de Diagnóstico Molecular, DASA, São Paulo, Brazil; 5 Divisão de Oncologia Clínica, Instituto do Câncer do Estado de São Paulo, Hospital das Clínicas da Faculdade de Medicina da Universidade de São Paulo, São Paulo, SP Brazil; 6 International Reasearch Center, A.C.Camargo Cancer Center, São Paulo, SP Brazil; 7 Department of Epidemiology, University Medical Center Groningen, University of Groningen, Groningen, The Netherlands

**Keywords:** Ovarian cancer, *BRCA1*, *BRCA2*, Next generation sequencing, MLPA

## Abstract

**Background:**

Approximately 8–15% epithelial ovarian cancer patients are *BRCA1* or *BRCA2* germline mutation carriers. Brazilian inhabitants may have peculiar genetic characteristics associated with ethnic diversity, and studies focusing on the entire *BRCA1/BRCA2* gene sequencing in Brazilian ovarian cancer patients are still lacking. The aim of this study was to evaluate *BRCA1/2* mutations, through entire gene sequencing, in a Brazilian population of women with epithelial ovarian cancer.

**Methods:**

In a cross sectional study performed in one reference centre for cancer treatment in São Paulo, Brazil, 100 patients diagnosed with epithelial ovarian cancer unselected for family history of breast and/or ovarian cancer were included. The complete coding sequence of *BRCA1*/2 genes was evaluated through Next-Generation or capillary sequencing. Large deletions were investigated through Multiplex Ligation-dependent Probe Amplification (MLPA).

**Results:**

Nineteen pathogenic mutations (*BRCA1*: *n* = 17 and *BRCA2*: *n* = 2) featuring 14 different mutations, including two large deletions in *BRCA1* (exon 1–2 deleted and exon 5–7 deleted) were identified. Three mutations were detected more than once (c.3331_3334delCAAG, c.5266dupC and c.4484G > T). Two novel frameshift mutations were identified, one in *BRCA1* (c.961_962delTG) and one in *BRCA2* (c.1963_1963delC). *BRCA1/2* mutations were seen in 35.5% of the patients with first and/or second-degree relatives with breast and/or ovarian cancer. Nineteen variants of uncertain significance (VUS) were detected (*BRCA1*: *n* = 2 and *BRCA2*: *n* = 17), including five distinct missense variants (*BRCA1*: c.5348 T > C; *BRCA2*: c.2350A > G, c.3515C > T, c.7534C > T, and c.8351G > A).

**Conclusions:**

Among epithelial ovarian cancer patients unselected for family history of cancer, 19% were *BRCA1/2* germline mutation carriers. Almost ¾ of the *BRCA* mutations, including two large deletions, were detected only once. Our work emphasizes the need of entire gene sequencing and MLPA screening in Brazil.

**Electronic supplementary material:**

The online version of this article (doi:10.1186/s12885-016-2966-x) contains supplementary material, which is available to authorized users.

## Background

Among the gynaecological malignancies, ovarian cancer has the highest mortality rate in developed countries and is the second leading cause of mortality in developing countries. In Brazil, 6150 new cases of ovarian cancer are expected in 2016, and in 2013, ovarian cancer accounted for 3283 deaths, indicating its importance in public health [1]. One feature that might contribute to this high mortality rate is that more than 60% of the patients are diagnosed in an advanced stage of the disease. [2].

One of the risk factors for ovarian cancer is germline mutations in *BRCA1* or *BRCA2* genes, accounting for approximately 8–15% of ovarian cancer cases worldwide [3–5]. Although the estimated cumulative incidence of ovarian cancer by the age of 70 is 40% (95% CI 35–46%) for *BRCA1* and 18% (95% CI 13–23%) for *BRCA2* mutation carriers [6], the precise risk estimates vary according to the population under study, ascertainment method and applied statistical technique [7].

Detection of *BRCA* mutation carriers may benefit both women who were already diagnosed with ovarian cancer, as well as their unaffected family members. These patients may benefit from platinum based chemotherapy [8] and PARP inhibitors [9], while unaffected family members may benefit from genetic counselling on risk reducing surgery, such as salpingo-oophorectomy, which may reduce their chances of developing ovarian cancer by 90% [10].

Although some studies evaluated *BRCA1*/2 mutations in breast and/or ovarian cancer patients in our country, only a few of them have performed the entire gene sequencing, none specifically in ovarian cancer patients [11–13]. Besides that, for most women with ovarian cancer, neither gene sequencing nor genetic counselling is currently available. Hence, the purpose of our work was to screen the entire *BRCA1* and *BRCA2* genes in a series of patients with epithelial ovarian cancer unselected for age or family history for breast and/or ovarian cancer treated in Brazil.

## Methods

### Patients

Patients were accrued at Instituto do Câncer do Estado de São Paulo (ICESP) in São Paulo city, Brazil. São Paulo is the largest city in Brazil and its metropolitan area has around 18 million people [14]. ICESP integrates the Brazilian public health network (SUS, Sistema Único de Saúde), which is responsible for the health care of ¾ of our population, and is the largest reference centre for cancer treatment in Latin America [15].

This study was approved by the Institutional Ethics Committee (Comitê de Ética da Faculdade de Medicina da Universidade de São Paulo, reference number 132/12 and 172/13) and an informed consent was signed by each participant.

Patients with invasive epithelial ovarian cancer, who were undergoing treatment or follow-up in the period between October 2012 and February 2015 at ICESP, were invited to participate. Inclusion criteria were invasive epithelial ovarian cancer diagnosis in the period beginning in January 2009 until the end of the study. Data regarding the tumour characteristics was obtained from the patient files.

To characterize our patients, women were asked to report their family history of breast and ovarian cancer, birth place and ancestry (defined as place of origin of direct ancestors, until third degree). Ancestry was considered unknown if there was no information at all or if parents were born in Brazil and there was no further information for at least second-degree ancestors.

Concerning family history of breast and ovarian cancer, patients were asked to report information about first and second-degree relatives. When women were able to report on two or more female relatives (≥45 years) from both sides of the family, they were classified as having a complete informative family history.

### DNA extraction from mononuclear cells

Total DNA was extracted from 10 mL blood samples using Illustra Blood GenomicPrep Mini spin kit (GE/28-9042-64, GE Healthcare Life Science) following the instructions of the manufacturer.

### *BRCA1* and *BRCA2* entire gene sequencing

The coding regions and exon-intron boundaries of *BRCA1* and *BRCA2* genes were sequenced by Sanger sequencing (*n* = 39) or by Next-Generation Sequencing (NGS) (*n* = 63). As a matter of comparison, two samples were analysed by both techniques, and results from both of them revealed the same single nucleotide substitutions and microdeletions.

### Polymerase chain reaction (PCR) amplification and Sanger sequencing

The complete coding regions of *BRCA1* (U14680 or NM_7294.3) and *BRCA2* (U43746 or NM_000059.3), including 50–100 base pairs (bp) of non-coding sequences, flanking the 5’ and 3’ ends of each exon, were amplified by PCR using 33 pairs of primers for *BRCA1* gene and 48 pairs of primers for *BRCA2* gene previously employed by other authors (Additional file 1). All pathogenic mutations were confirmed through a second Sanger sequencing.

### Next-generation sequencing

For the NGS analysis, the Ion AmpliSeq™ *BRCA1* and *BRCA2* Panel (Life technologies) was used. The panel consists of three primer pools (167 amplicons) that target the entire coding regions, including 10–20 bp of non-coding sequences, flanking the 5’ and 3’ ends of each exon. Samples were sequenced on a 314 v2 Ion Chip taking 12 samples per chip using a Personal Genome Machine (PGM) sequencer (Ion Torrent™), and the Ion PGM Sequencing 200 Kit version 2 (Life Technologies). Data analysis consisting of annotation of single-nucleotide variants, insertions, deletions, and splice site alterations was performed using the Ion Reporter™ Server System (Life Technologies). Sequence data were also visually evaluated through Integrative Genomics Viewer (IGV). If a nucleotide had coverage under 50x, the affected region was re-sequenced by Sanger methodology. In addition, all pathogenic variants were re-sequenced by Sanger methodology for confirmation. Full details of methods are given in the Additional file 1.

### Multiplex ligation-dependent probe amplification

All patients had their DNA investigated for large rearrangements of *BRCA1* and *BRCA2* genes, specifically deletions and duplications, and *CHEK2* (c.1100delC) point mutation, through the Multiplex Ligation-dependent Probe Amplification (MLPA) methodology (*BRCA1:* SALSA® MLPA® P002 *probemix*; *BRCA2*: SALSA® MLPA® P045 *BRCA2*/CHEK2 probemix; MRC-Holland, Amsterdam, The Netherlands), as described on Additional file 1. Patients showing positive results had DNA samples analysed by a different set of MLPA probemix (*BRCA1*: SALSA® MLPA® P087; *BRCA2*: SALSA® MLPA® P077, MRC-Holland, Amsterdam, The Netherlands).

### Classifications of variants

All sequence variants were named according to the nomenclature proposed by the Human Genome Variation Society, HGVS [16]**.** *BRCA1* and *BRCA2* variants were searched in five publicly accessible databases. Additionally, gene variants were submitted to *in silico* prediction models, PolyPhen-2 [17], SIFT [18], Align-GVGD [19], for missense variants; Provean [20] for in-frame deletions, and Human Splicing Finder [21] to check for intronic and exonic variants leading to potential splicing defects. Minor allele frequency was checked in the 1000 Genomes Project database [22], the Exome Aggregation Consortium (ExAC) [23], the Global MAF dbSNP [24], the Exome Variant Server, NHLBI GO Exome Sequencing Project (ESP) [25]. Minor allele frequency in probands was also evaluated (Additional file 1: Table S2-S3).

The variants were then classified according to recommendations of the American College of Medical Genetics and Genomics in: pathogenic, likely pathogenic, benign, likely benign and variant of uncertain significance (VUS) [26]. Variants for *BRCA1* were also checked for co-occurrence with known pathogenic mutations in the same patient. When the VUS were present in two or more databases and classified as benign (BIC and ClinVar), not affect function (LOVD), 1-not pathogenic (LOVD-IARC), 1-neutral (UMD/BRCA Share™) they were considered as benign on the present analysis.

## Results

One hundred women were included, with a median age at the time of diagnosis of 55.0 years (33–81 years) and at time of inclusion in this study of 56.5 years (34–81 years). The median interval between date of diagnosis and date of inclusion in the study was 18 months (1–54 months). Most patients were diagnosed with serous adenocarcinoma (84%) and advanced stage disease (clinical stages III/IV; 78%), six patients had a prior diagnosis of breast cancer (Additional file 1: Table S1). Most women were born in the Southeast region of Brazil (57%), mainly in Sao Paulo state (48%), including those born in Sao Paulo city (25%); 28% of the patients were born in the Northeast region of the country, mainly in Bahia state (12%) (Table 1). Concerning ancestry, 38% of the patients reported Brazilian only ancestors, 14% and 5% reported European only or Japanese only ancestors in both sides of the family, respectively. In addition, 7% of the patients reported at least one indigenous ancestor, in concomitance with Brazilian and/or European ancestries (Table 1). Thirty-two out of 100 patients were not able to provide a complete informative family history; 31 of the remaining 68 patients reported at least one first and/or second-degree relative with breast and/or ovarian cancer.

**Table 1:** Clinical and Pathological features of ovarian cancer patients according to deleterious *BRCA1/2* mutations

	*BRCA 1/2* mut	*BRCA1/2* wt
	*n* = 19	*n* = 81
Age at diagnosis, median (range), years	54 (39–63)	55 (33–81)
Time between diagnosis and inclusion (months)	23.33 ± 14.53	19.83 ± 13.52
Histology, *n* (%)		
Serous	18 (94.7)	66 (81.5)
Non-serous	1 (5.3)	15 (18.5)
Clinical Stage, *n* (%)		
I/II	0 (0)	21 (25.9)
III/IV	19 (100)	59 (72.8)
Missing	0 (0)	1 (1.2)
Region of origin, *n* (%)		
Southeast	9 (47.4)	48 (59.3)
Northeast	7 (36.8)	21 (25.9)
South	2 (10.5)	8 (9.9)
North	1 (5.3)	1 (1.2)
Abroad	0 (0)	2 (2.4)
Missing	0 (0)	1 (1.2)
Ancestry until 3^rd^ degree, *n* (%)		
Brazilian only	7 (36.8)	31 (38.3)
European only	2 (10.5)	12 (14.8)
Japanese only	1 (5.3)	4 (4.9)
Asian only	0 (0)	1 (1.2)
Brazilian and European	5 (26.3)	16 (19.8)
Brazilian and Indigenous	2 (10.5)	1 (1.2)
Indigenous and European	0 (0)	1 (1.2)
Indigenous and Brazilian and European	2 (10.5)	1 (1.2)
Brazilian and African	0 (0)	2 (2.5)
Brazilian and African and European	0 (0)	1 (1.2)
Brazilian and Japanese	0 (0)	2 (2.5)
Unknown	0 (0)	8 (9.9)
Missing	0 (0)	1 (1.2)
Family history for breast and/or ovarian cancer		
Positive (first and/or second-degree relatives)	11 (57.9)	20 (24.7)
Negative	5 (26.3)	32 (39.5)
Not Completely Known/Unknown	3(15.8)	29 (35.8)

Pathogenic mutations in *BRCA1* and *BRCA2* genes were detected in 19 patients, 17 in *BRCA1* and two in *BRCA2* (Table 2; Additional file 1: Table S2-S3). Mutations in *BRCA1*, comprised five different frameshift mutations, two of which were present in three different patients (c.3331_3334delCAAG and c.5266dupC) and one detected for the first time in the current study (c.961_962delTG). *BRCA1* mutations also included two nonsense mutations in exon 11; one missense mutation (c.4484G > T, detected in two patients) and two splice site variants (c.4675 + 1G > A, in exon 15, and c.5074 + 2 T > C, in exon 17).

**Table 2:** *BRCA1/2* mutations in ovarian cancer patients: clinical aspects and molecular description

ID	HGVS cDNA	HGVS protein	BIC name	Type	Age	Ancestry	HT	CS	FH	Reference
*BRCA1*										
21	c.791_794delGTTC	p.Ser264Metfs	910del4	F	50–59	BRZ	S	III	NC	[30]
18	c.961_962delTG	p.(Trp321fs)	ND	F	50–59	BRZ	S	III	-	Current Study
49	c.1687C > T	p.Gln563Ter	1806C > T	N	40–49	BRZ	S	III	+	[30]
39	c.2215A > T	p.Lys739Ter	K739X	N	50–59 (BC ≤50)	BRZ	S	III	NC	[BIC]
17	c.3331_3334delCAAG	p.Gln1111Asnfs	3450del4	F	30–39	IND/BRZ/EUR	S	III	+	[5, 11, 13, 30]
42	c.3331_3334delCAAG	p.Gln1111Asnfs	3450del4	F	50–59	IND/BRZ/EUR	S	III	+	[5, 11, 13, 30]
107	c.3331_3334delCAAG	p.Gln1111Asnfs	3450del4	F	50–59 (BC ≤50)	BRZ/EUR	E	III	+	[5, 11, 13, 30]
50	c.4484G > T	p.Arg1495Met	R1495M	M	30–39	BRZ	S	III	-	[4]
37	c.4484G > T	p.Arg1495Met	R1495M	M	50–59	IND/BRZ	S	IV	+	[4]
106	c.4675+1G > A	-	IVS15+1G >A	Ss	40–49	BRZ/EUR	S	III	-	[4]
67	c.5074+2 T > C	-	IVS17+2 T >C	Ss	60–69	BRZ/EUR	S	III	+	[BIC]
85	c.5098_5098delA	p.Thr1700Hisfs	5217delA	F	40–49	BRZ/EUR	S	IV	-	[BIC]
26	c.5266dupC	p.Gln1756Profs	5382insC	F	50–59	EUR	S	IV	+	[11, 13, 32]
56	c.5266dupC	p.Gln1756Profs	5382insC	F	50–59 (BC ≤50)	BRZ	S	III	+	[11, 13, 32]
108	c.5266dupC	p.Gln1756Profs	5382insC	F	40–49	IND/BRZ	S	III	UK	[11, 13, 32]
101	Exon 1–2 deleted^a^	-	-	LGR	50–59	BRZ	S	III	+	[37, 38]
9	Exon 5–7 deleted^a^	-	-	LGR	50–59	BRZ /EUR	S	III	-	[37, 38]
*BRCA2*										
74	c.1963_1963delC	p.(Pro655fs)	ND	F	60–69	EUR	S	III	+	Current Study
102	c.5576_5579delTTAA	p.Ile1859_Lys1860?fs	5804del4	F	60–69	JAP	S	III	+	[30]

*ID* patient identification; Type*: F* frameshift, *N* nonsense, *M* missense, *Ss* splice site, *LGR* large genomic rearrangement; Age in years (range); *BC* previous breast cancer history; Ancestry: *BRZ* Brazilian, *IND* Indigenous, *EUR* European, *JAP* Japanese, *CS* clinical stage, *HT* histological type, *S* serous, *E* endometrioid, *FH* family history for breast and/or ovarian cancer: (+): present, (−): absent, *NC* not completely known, *UK* unknown, *BIC* breast cancer information core, *ND* not described mutation

^a^Indicates mutations detected by MLPA

*BRCA1* rearrangements were identified in two patients (Table 2). One patient presented one large deletion involving exons 5, 6 and 7, detected by two different sets of MLPA probemix (SALSA® MLPA® P002 and SALSA® MLPA® P087). Another patient displayed an inconclusive result using SALSA® MLPA® P002 *BRCA1* probemix, which provided questionable low signals for the 1a, 1b and 2 exons repeatedly, probably due to incomplete denaturation of the CpG islands near these exons. A confirmatory MLPA reaction with a second set of probes (SALSA® MLPA® P087 *BRCA1* probemix) corroborated the presence of this rearrangement.

Pathogenic mutations in *BRCA2*, both frameshift, were detected in only two patients, one of them identified for the first time in the present study (c.1963_1963delC, exon 11). A suspected deletion of *BRCA2* exon 15 was at first detected, but was not confirmed. This patient harboured a missense variant (c.7534C > T; variant of uncertain significance) localized within one of the *BRCA2* probes of SALSA® MLPA® P045, which may have interfered in the result and reduced the signal of exon 15. A confirmatory MLPA reaction, using SALSA® MLPA®P077, employing different probes, did not reveal a reduced peak for exon 15, indicating the absence of a true deletion.

All patients were checked for c.1100delC point mutation of CHEK2, however, none was detected.

There was no difference between the median age at diagnosis from patients who were *BRCA1/2* mutation carriers or non-carriers. All mutation carriers were diagnosed with advanced disease (clinical stages III/IV). Considering the birth place, nine out of 57 women (15.8%) born in the Southeast region and seven out of 28 (25%) born in the Northeast region harboured a *BRCA1/2* mutation. Among 38 patients who reported Brazilian only ancestry, seven were *BRCA1/2* mutation carriers (18.4%) and among seven patients who reported at least one indigenous ancestry four (57.1%) were mutation carriers (Table 1). Among the 68 patients with informative family history for breast and/or ovarian cancer, 35.5% with and 13.5% without any affected first and/or second-degree relatives were *BRCA1/2* mutations carriers. Although all two patients who were *BRCA2* mutation carriers were aged at least 60 years, almost all (16/17) women who were *BRCA1* mutation carriers were aged less than 60.

Nineteen variants of uncertain significance (VUS) were detected, two in *BRCA1* and seventeen in *BRCA2* gene. Among the VUS, five distinct missense variants were identified, one in *BRCA1* and four in *BRCA2*, among which, three, *BRCA1* c.5348 T > C*, BRCA2* c.3515C > T and *BRCA2* c.8351G > A were predicted deleterious in at least three of four mutation function prediction models (Polyhen-2, SIFT, Provean or Align GVGD) (Table 3). The remaining VUS were synonymous (*n* = 4) or were located in intronic regions, at least eight nucleotides away from the intron-exon boundary (*n* = 10).

**Table 3:** Analysis of missense variants from *BRCA1/2* gene of uncertain significance using mutation function prediction models

Gene	Exon	HDVS cDNA	HGVS protein	PolyPhen	SIFT	Provean	Align GVGD	Human splice finder	ID
*BRCA1*	22	c.5348 T > C	p.Met1783Thr	Probably Damaging	Damaging	Neutral	Class C45	-	94
*BRCA2*	11	c.2350A > G	p.Met784Val	Benign	Tolerated	Neutral	Class C0	Activation of an exonic cryptic donor site / Creation of an exonic ESS site / Alteration of an exonic ESE site.	35
	11	c.3515C > T	p.Ser1172Leu	Probably Damaging	Damaging	Deleterious	Class C0	Creation of an exonic ESS site/Alteration of an exonic ESE site.	25
	15	c.7534C > T	p.Leu2512Phe	Probably Damaging	Damaging	Neutral	Class C0	Alteration of an intronic ESS site.	47
	19	c.8351G > A	p.Arg2784Gln	Probably Damaging	Damaging	Neutral	Class C35	Activation of an exonic cryptic acceptor site	111
								Presence of one or more cryptic branch point.	

*HGVS* Human Genome Variation Society, PolyPhen: Variant Benign, Possibly damaging and Probably damaging; SIFT: Variant Tolerated (benign) or Damaging; Provean: Neutral or Deleterius; Align GVGD: Class C0 (Less likely to interfere in protein function), C15, C25, C35, C45, C55, C65 (More likely to interfere in protein function); ID: patient identification

## Discussion

In this cohort, 19 out of 100 unselected Brazilian ovarian cancer patients, were *BRCA1/2* mutation carriers, mainly in the *BRCA1* gene (*n* = 17). In *BRCA1* gene, 12 different mutations were detected, including one new frameshift and two large deletions. In *BRCA2* gene, only two pathogenic mutations, including a new frameshift mutation were found. In addition, another five missense VUS were identified in five different patients. Concerning their ancestry, 18.4% of the patients who reported Brazilian only ancestors and 26.3% of those who reported European ancestry, in at least one side of the family, were mutation carriers. In addition, 35.5% of the patients with and 13.5% of the patients without first and/or second-degree relatives with breast and/or ovarian cancer were *BRCA1/2* mutations carriers.

A prevalence of 19% *BRCA1*/2 mutation rate seems somewhat higher than previously described for women with ovarian cancer from other countries, such as Canada (13.4%) [4] and Colombia (15%) [5]. However, one might claim that we had a cohort enriched in *BRCA* mutation carriers, due to time selection, taking into consideration that germline mutation in *BRCA1* or *BRCA2* is associated with an improved 5-year overall survival and that a few patients were diagnosed long before their enrolment in the project [27, 28]. Contrary to this is the fact that the mean time between dates of diagnosis and enrolment was similar in both *BRCA1*/2 mutation and *BRCA1*/2 wild type carriers.

In accordance with previous studies, our patients with ovarian cancer were mainly *BRCA1* mutation carriers [4], however only one out of 12 different mutations, c.2215A > T, was located in a putative ovarian cancer cluster region (OCCR) of exon 11, an hypothesized region associated with increased risk estimates for ovarian cancer [29]. Remarkably was that this patient had breast cancer before the ovarian cancer.

Three mutations in *BRCA1* gene were detected more than once: c.3331_3334delCAAG, c.5266dupC and c.4484G > T. *BRCA1* c.3331_3334delCAAG mutation in exon 11, is also a frequent mutation in ovarian cancer patients from Colombia [5] and Spain [30], but mostly rare in Canadian patients [4]. This mutation was already reported in Brazilian patients [11, 13]. A previous haplotype study suggested that it could represent a founder effect of Spanish origin [31], and curiously all our three patients reported European ancestries, two of them from the Iberian Peninsula, more specifically, from Portugal.

Another frequent mutation detected in our ovarian cancer patients was the one commonly found in the Ashkenazi Jewish, *BRCA1* c.5266dupC (5382insC), however none of them reported Jewish ancestry. This mutation is one of the most commonly found in Brazilian breast and/or ovarian cancer patients [11, 13, 32]. In addition, one meta-analysis reported that *BRCA1* c.5266dupC is the fourth most prevalent in Latin America [33]. Interestingly, a haplotype study revealed that *BRCA*1 c.5266dupC originated from a single common ancestor around 1800 years ago in northern Russia and spread to various populations, including Ashkenazi Jewish people [34]. In accordance, a common origin for this mutation in our country was previously reported for breast cancer patients [35, 36].

Two patients presented the *BRCA1* c.4484G > T missense mutation, which involves the last nucleotide of exon 14, resulting in skipping of exon 14. This mutation was previously found in ovarian cancer patients and associated with causality [4]. Other mutations in *BRCA1* detected in the present work were already described, as shown in Table 2, except for c.961_962delTG frameshift variant, characterized for the first time in this study.

Comparing *BRCA1* mutations detected in our study of Brazilian ovarian cancer patients with those reported in breast cancer patients [11–13], it is interesting to observe that they are concordant with respect to the most recurrent variant, *BRCA1* c.5266dupC, that also is one of the most commonly found in Latin American breast cancer patients [33]. However, there is a difference in relation to variant c.3331_3334delCAAG, that was rarely found in patients with breast cancer.

Only two pathogenic mutations were detected in *BRCA2* gene, including one novel frameshift mutation, c.1963_1963delC, and another one, c.5576_5579delTTAA, in a patient with Japanese ancestry. The latter mutation is located in an ovarian cancer cluster region (OCCR) of exon 11, which is bound by c.3249 and c.5681 [29].

Another mechanism of gene inactivation, namely the rearrangement of large tracts of genomic DNA, was detected in two patients (*BRCA1:* exon 1–2 deleted and exon 5–7 deleted). Deletion of exons 1a-2, which may affect production and/or stability of the transcript [37, 38], is the third most frequent in Latin American breast cancer patients [33]. In your study, this deletion was identified in a woman who was diagnosed with ovarian cancer at the age of 52 and reported a family history of ovarian cancer and colorectal cancer. The removal of exons 5–7 from gene *BRCA1* causes a frameshift in protein translation [37]. This deletion was identified in a woman with ovarian cancer aged 57 years, who reported an European ancestry but did not recall any family history of breast and/or ovarian cancer. This mutation was previously described in one German patient with positive family history for breast cancer and ovarian cancer [37] and one Italian patient with breast cancer [38].

In our study, *CHEK2* mutations were not detected, in accordance with other previous studies, that in total analysed an additional 21 Brazilian ovarian cancer patients. [12, 39].

A strength of our study is that the entire *BRCA1/2* gene was screened for the first time in a population of women with epithelial ovarian cancer from Brazil, unselected for age and family history. A weakness however, is the small sample size was analysed. In a recently published study, the prevalence of a panel of eight mutations in *BRCA1* or *BRCA2* genes, mainly detected in Ashkenazi Jewish or people from Russia or Poland, was investigated in 106 ovarian cancer patients treated at the Federal Hospital in Minas Gerais state, which is neighbour to São Paulo state. None of the participants were found to carry any of the genotyped mutations [40]. In our study, among nine patients born in Minas Gerais state, one harboured a *BRCA1* mutation (c.4675 + 1G > A) not investigated in the previous study (Table 2). In addition, in another study performed in our city, a few patients with ovarian cancer were enrolled for entire BRCA1/2 gene sequencing. *BRCA1* mutation was detected in one out of three patients diagnosed with ovarian cancer and in four out of nine with both breast and ovarian cancer [12].

Considering that Brazil is a huge country inhabited by people from different origins and that our patients come from different regions of the country, we evaluated *BRCA1/2* mutation status considering their birth place and ancestry. Our study mainly reflects Brazilian patients born in São Paulo state or in the Northeast region of Brazil with Brazilian and/or European ancestries. In the current study 14 different variants were detected in 100 patients. These different variants may reflect the ethnic diversity and miscegenation of people that live in Brazil. Corroborating this hypothesis, genetic polymorphisms analysis have already revealed that the average Brazilian population has unique characteristics, comprehending a mixture of European, African and Amerindian ancestry genes [41]. It is interesting to observe that, 15 out of 16 *BRCA1* mutation carriers, reported Brazilian ancestry in one or both sides of the family, which means that, as long as they were aware, their ancestors were born in Brazil.

## Conclusion

In this cohort of epithelial ovarian cancer patients, the prevalence of *BRCA1/2* mutation was 19%, mostly detected in different gene locations. Two novel frameshift mutations were identified, one in *BRCA1* and one in *BRCA2*, as well as two large deletions. These data emphasize that entire gene sequencing of both *BRCA1* and *BRCA2* as well as MLPA screening should be offered to all Brazilian ovarian cancer patients.

## Additional file

Additional file 1: Supplementary Methods. **Table S1.** Clinical and pathological characteristics, *BRCA* sequencing and MLPA results. **Table S2.** *BRCA1* gene variants. **Table S3.** *BRCA2* gene variants. (ZIP 73.7 kb)

## Abbreviations

Align-GVGD: Grantham Variation (GV) which measures the degree of biochemical variation among amino acids found at a given position in the multiple sequence alignment, Grantham Deviation (GD), which reflects the ‘biochemical distance’ of the mutant amino acid from the observed amino acid at a particular position.
BIC: Breast cancer information core
bp: Base pairs
dbSNP: The single nucleotide polymorphism database
DNA: Deoxyribonucleic acid
ESP: Exome sequencing project
ExAC: The exome aggregation consortium
HGVS: Human Genome Variation Society
ICESP: Instituto do Câncer do Estado de São Paulo
IGV: Integrative genomics viewer
LOVD: Leiden open variation database
LOVD-IARC: Leiden open variation database - The International Agency for Research on Cancer
MAF: Minor allele frequency
MLPA: Multiplex ligation-dependent probe amplification
NGS: Next-generation sequencing
OCCR: Ovarian cancer cluster region
PARP: Poly (ADP-ribose) polymerase
PCR: Polymerase chain reaction
PGM: Personal genome machine
PolyPhen-2: Polymorphism Phenotyping v2
Provean: Protein variation effect analyzer
SIFT: Sorting intolerant from tolerant
SUS: Sistema Único de Saúde (Brazilian Public Health System)
UMD: Universal mutation database
VUS: Variant of uncertain significance
