# Supplementary material for: Germline mutations in BRCA1 and BRCA2 in epithelial ovarian cancer patients in Brazil
Source: BMC Cancer. 2016 Dec 3;16:934. doi: 10.1186/s12885-016-2966-x (PMC5135756; doi:10.1186/s12885-016-2966-x)
Supplement: Additional file 1: — Supplementary Methods. Table S1. Clinical and pathological characteristics, BRCA sequencing and MLPA results. Table S2. BRCA1 gene variants. Table S3. BRCA2 gene variants. (ZIP 73.7 kb) [file 12885_2016_2966_MOESM1_ESM.zip › Supplementary methods.docx]

**Supplementary Methods**

***DNA extraction from mononuclear cells***

Total DNA was extracted from 10 mL blood samples using Illustra Blood GenomicPrep Mini spin kit (GE/28-9042-64, GE Healthcare Life Science) following the instructions of the manufacturer. The DNA concentration and purity was determined using the Nano Drop 1000 spectrophotometer (Thermo Fisher Scientific, Massachusetts, USA), the 260/280 and 260/230 ratios varied from 1.6 to 2.1 and 1.8 to 2.3, respectively. DNA concentration from samples analysed by next generation sequencing were also evaluated using a Qubit® dsDNA BR Assay kit in a Qubit® 3.0 Fluorometer (Life Technologies).

***Polymerase chain reaction (PCR) Amplification and Sanger Sequencing***

The complete coding regions of *BRCA1* (U14680 or NM_7294.3) and *BRCA2* (U43746 or NM_000059.3), including 50-100 base pairs (bp) of non-coding sequences, flanking the 5’ and 3’ ends of each exon, were amplified by PCR using 33 pairs of primers for *BRCA1* gene [1-2] (Additional Table 1) and 48 pairs of primers for *BRCA2* gene [3] (Additional Table 2), and sequenced in both forward and reverse directions. AmpliTaq Gold enzyme 250U (final concentration 0,4U/µl) (Applied Biosystems, Foster City, CA, USA), 1X AmpliTaq Gold buffer, 1.5-3.0 mM AmpliTaq Gold MgCl2, 0.16 mM dNTP (Applied Biosystems, Foster City, CA, USA), 0.4 µL of each primer and 50 ng of DNA in a total volume of 10 µL in a GeneAmp 9700 Thermal Cycler (Applied Biosystems, Foster City, CA, USA). The PCR cycling consisted of 1 cycle at 95°C for 10 min, 40 cycles at 94°C for 50 sec, 54- 66°C for 50 sec, 72°C for 50 sec, and 1 cycle at 72°C for 7 min. *BRCA2* exon 11 fragments were amplified by touchdown PCR, with annealing temperatures decreasing from 63°C to 56°C for fragments corresponding to the beginning of this exon to nucleotide 4526, and annealing temperatures from 68°C to 61°C for fragments corresponding to the ending of the exon. PCR products were loaded onto a 1,5% agarose gel, stained with GelRed Nucleic Acid Stain (Biotium, Hayward, CA, USA), and evaluated.

Each PCR product was then treated with 1 µL of illustra™ ExoStar™ 1-Step (GE Healthcare Life Sciences) and incubated at 37°C for 15 minutes followed by 80°C for 15 minutes. All PCR products were sequenced in both forward and reverse directions using BigDye® Terminator v3.1 (Life Technologies or Applied Biosystems) according to the manufacturer’s instructions, and were analyzed on a 3500 Genetic Analyzer (Applied Biosystems - Hitachi). Sequences obtained were visualized by Chromas v2.33 (Technelysium Pty. Ltd.) and by Mutation Surveyor v3.20 (SoftGenetics LLC). If a mutation was identified, a new sample was collected from the same patient and was sequenced to confirm the result.

***Next-Generation Sequencing***

For the NGS analysis, the Ion AmpliSeq™ *BRCA1* and *BRCA2* Panel (Life technologies) containing 167 primer pairs in three pools was used. Library preparation was performed using the Ion AmpliSeq™ Library Kit 2.0 protocols. The DNA amplification was carried out using 30 ng of DNA and with of the three primer pools and the 5x Ion AmpliSeq™ HiFi Master Mix in a Verity Thermal Cycler (Thermo Fisher Scientific) for 2 min at 99°C, followed by 19 cycles of 99°C for 15 sec and 60°C for 4 min, ending with a holding period at 10°C. Afterwards, the three resultant PCR amplicons were mixed (30 µL), and 20 µL were treated with 2 µL FuPa reagent to partially digest primer sequences and phosphorylate the amplicons at 50°C for 10 min, followed by 55°C for 10 min, then 60°C for 20 min. In the next step, sequencing adaptors (A: conjugated to biotin and P1) and barcodes (consisting of short stretches of index sequences that enable sample multiplexing) were ligated to the amplicons using the Ion Xpress™ Barcode Adapters kit (Life Technologies) which contain a ligase and cofactor, for 30 min at 22°C then 72°C for 20 min. The Adaptor-ligated amplicon (libraries) were purified using 45 μL (1.5X sample volume) of Agencourt® AMPure® XP Reagents (Beckman Coulter) to each library and were incubated for 5 minutes at room temperature. The tube were placed in a magnetic rack such and were incubated for 2 minutes or until solution clears. After, the supernatant was removed carefully and discarded without disturbing the pellet, the freshly prepared 70% ethanol (150 μL) was added and the tube was moved side‐to‐side in the two positions of the magnet to wash the beads, and then the supernatant was removed and discarded (two rounds of purification were repeated). The tube was maintained in the magnet, the beads were air‐dried at room temperature for 5 minutes. The library was subjected to the second round of amplification using 50 μL of Platinum® PCR SuperMix High Fidelity and 2 μL of Equalizer™ Primers to each bead pellet for 2 min at 98°C, followed by 7 cycles of 98°C for 15 sec and 64°C for 1 min, ending with a holding period at 10°C in a Verity Thermal Cycler. After thermal cycling, 10 μL of Equalizer™ Capture were added to each library amplification reaction and were mixed by pipetting and incubated at room temperature for 5 minutes. Next, 6 μL of washed beads were added to each tube containing the capture reaction, were mixed and incubated at room temperature for 5 minutes. After then, the tube was placed in the magnet and incubated for 2 minutes or until the solution is clear. After, the supernatant was removed carefully and discarded without disturbing the pellet, the Equalizer™ Wash Buffer (150 μL) was added to each reaction, and was moved side‐to‐side in the two positions of the magnet to wash the beads, and then the supernatant was removed and discarded (two rounds of purification were repeated). With the plate still in the magnet, the supernatant were carefully removed and discarded without disturbing the pellet (twice). Next, the tube was removed from the magnet and was added 100 μL of Equalizer™ Elution Buffer to each pellet, were mixed and incubated in a thermal cycler at 32°C for 5 minutes. The tube was placed in the magnet and incubated at room temperature for 5 minutes or until the solution is clear. The supernatant contains the equalized library at ~100 pM, which were combined (12 samples). Next, emulsion PCR was carried out using the Ion OneTouch™ System and Ion OneTouch™ 200 Template Kit v2 (Life Technologies), the Template-positive Ion Sphere™ Particles (ISPs) were then enriched with Dynabeads MyOne™ Streptavidin C1 Beads (Life Technologies) using an Ion OneTouch™ ES System (Life Technologies). The quality of the ISPs was assessed using a Qubit 2.0 Fluorometer (Life Technologies). Enriched ISPs were subjected to sequencing on a 314 v2 Ion Chip (12 samples per chip) using a Personal Genome Machine (PGM) sequencer (Ion Torrent™) and the Ion PGM Sequencing 200 Kit version 2 (Life Technologies). Sequencing was performed using 500 flow runs that generated approximately 200 bp. The PGM sequencing run outputs were directly loaded to the Torrent Server and stored as ‘.dat’ files.

Data analysis consisting of annotation of single-nucleotide variants, insertions, deletions, and splice site alterations was performed by the Ion Reporter™ Server System (Life Technologies. Sequence data were also visually evaluated through Integrative Genomics Viewer (IGV).

Sequences with coverage under 50x the corresponding genomic region was checked by Sanger methodology, the same for the pathogenic variants and new variants.

***Multiplex Ligation-Dependent Probe Amplification (MLPA) of BRCA1 and BRCA2 Genes***

All Patients were investigated for large rearrangements, specifically deletions and duplications, using the Multiplex ligation-dependent probe amplification (MLPA) commercial kits: *SALSA® MLPA® P002 BRCA1 probemix* (P002-100R), and SALSA® MLPA® P045 *BRCA2*/CHEK2 probemix (P045-100R) (MRC-Holland, Amsterdam, The Netherlands). Patients showing positive results were then analysed by different set of MLPA probemix (*BRCA1*: SALSA® MLPA® P087; *BRCA2*: SALSA® MLPA® P077, MRC-Holland, Amsterdam, The Netherlands).

At first, 50 ng of genomic DNA in 2,5 µl ultrapure water was denaturated for ten minutes at 98°C after which 1,5 μl of the probemix mixture was added (0,75 µl de sonda MLPA e 0,75 µl de MLPA buffer). The sample DNA and probemix mixture were heated at 95°C for 1 minute and incubated at 60°C overnight (17 h). Afterwards, ligation was performed with 1,5 µl of ligase buffer A, 1,5 µl of ligase buffer B, 0,5 µl of Ligase-65 and 12,5 µl of water and maintained at 54°C for 15 minutes. Then ligase was inactivated by incubation for five minutes at 98°C. Amplification was performed by adding 5 µl Mix Polimerase (1 µl de Salsa PCR primers, 0,25 µl of Salsa polimerase and 3,75 µl water) and heated at 95°C for 1 minute. PCR was carried out for 35 cycles (30 sec at 95°C, 30 sec at 60°C and 60 sec at 72°C) followed by 20 minutes at 72°C in a GeneAmp 9700 Thermal Cycler (Applied Biosystems). Afterwards, was using 1 µl of PCR product diluted 1:10 in water, 0,075 µl GeneScan™ 600 LIZ® dye Size Standard v2.0 (*Applied Biosystems* - 4408399) and 9 µl HiDi Formamida (*Thermo Fisher Scientific* [4440753](https://www.thermofisher.com/order/catalog/product/4440753)) and incubated at 80°C for 2 minutes in a GeneAmp 9700 Thermal Cycler (Applied Biosystems). For normalizing the data, at least three genomic DNA samples obtained from peripheral blood cells of health donors were always run as controls in each analysis. The fragments were analyzed on an Applied Biosystems 3500 Genetic Analyzer (Applied Biosystems - Hitachi) and analysis was performed using Coffalyser.NET software (MRC-Holland, Amsterdam, Netherlands). Normal values were considered when ratio was between 0.8 and 1.2.

***Classifications of variants***

All sequence variants were named according to the nomenclature used by the Human Genome Variation Society, HGVS [4]. *BRCA1* and *BRCA2* variants were searched in five publicly accessible databases: the Breast Cancer Information Core [5], the Leiden Open Variation Database [6], the Leiden Open Variation Database – International Agency for Research on Cancer [6], the *BRCA1* and *BRCA2* Universal Mutation Database (UMD)[8], and the ClinVar [9], freeze November, 2015. Additionally, gene variants were submitted to *in silico* prediction models, such as Polymorphism Phenotyping v2 (PolyPhen-2) [10], Sorting Intolerant From Tolerant (SIFT) [11], Align-GVGD [12], for missense variants; Protein Variation Effect Analyzer (Provean) [13] for in-frame deletions, and Human Splicing Finder [14] to check for intronic and exonic variants leading to potential splicing defects. Minor allele frequency was determined from the 1000 Genomes Project database [15], the Exome Aggregation Consortium (ExAC) [16], the Global MAF dbSNP [17], the Exome Variant Server, NHLBI GO Exome Sequencing Project (ESP), [18].

The variants were then classified according to recommendations of the American College of Medical Genetics and Genomics in: pathogenic, likely pathogenic, benign, likely benign and variant uncertain significance (VUS) [19]. Variants for *BRCA1* were also checked for co-occurrence with known pathogenic mutations in the same patient. All VUS were revalued and when present in two or more databases and classified as benign (BIC and ClinVar), no affect function (LOVD), 1-not pathogenic (LOVD-IARC), 1-neutral (UMD) they were reclassified in our analysis, as benign.

***References***

[1] Arnold N, Gross E, Schwarz-Boeger U, Pfisterer J, Jonat W, Kiechle M. A highly sensitive, fast, and economical technique for mutation analysis in hereditary breast and ovarian cancers. Hum Mutat. 1999;14(4):333-9.

[2] Friedman LS, Ostermeyer EA, Szabo CI, Dowd P, Lynch ED, Rowell SE, et al. Confirmation of BRCA1 by analysis of germline mutations linked to breast and ovarian cancer in ten families. Nat Genet. 1994;8(4):399-404.

[3 Wagner TM, Hirtenlehner K, Shen P, Moeslinger R, Muhr D, Fleischmann E, et al. Global sequence diversity of BRCA2: analysis of 71 breast cancer families and 95 control individuals of worldwide populations. Hum Mol Genet. 1999;8(3):413-23.

[4] Human Genome Variation Society, HGVS. <http://www.hgvs.org>. Accessed 01 Oct 15.

[5] Szabo C, Masiello A, Ryan JF, Brody LC. The breast cancer information core: databases design, structure, and scope. Hum Mutat. 2000;16:123-31. Breast Cancer information Core (BIC) databases. http://research.nhgri.nih.gov/bic/. Accessed Nov 15.

[6] Fokkema IF, Taschner PE, Schaafsma GC, Celli J, Laros JF, den Dunnen JT. LOVD v.2.0: the next generation in gene variant databases. Hum Mutat. 2011;32:557-63.

[7] Leiden Open variation -International Agency for Research on Cancer (LOVD-IARC) Databases. <http://hci-exlovd.hci.utah.edu/variants.php?select_db=BRCA1&action=view_all>; <http://hci-exlovd.hci.utah.edu/home.php?select_db=BRCA2>. Accessed 31 Nov 15.

[8] Caputo S, Benboudjema L, Sinilnikova O, Rouleau E, Béroud C, Lidereau R; French BRCA GGC Consortium. Description and analysis of genetic variants in French hereditary breast and ovarian cancer families recorded in the UMD-*BRCA1*/*BRCA2* databases. [Nucleic Acids Res. 2012 Jan;40 (Database issue):D992-1002.](http://www.ncbi.nlm.nih.gov/pubmed/22144684?ordinalpos=1&itool=EntrezSystem2.PEntrez.Pubmed.Pubmed_ResultsPanel.Pubmed_DefaultReportPanel.Pubmed_RVDocSum)

[9] ClinVar Databases. [http://www.ncbi.nlm.nih.gov/clinvar/?term=BRCA1%5Bgene %5D](http://www.ncbi.nlm.nih.gov/clinvar/?term=BRCA1%5Bgene%20%5D); <http://www.ncbi.nlm.nih.gov/clinvar/?term=BRCA2%5Bgene%5D>. Accessed 31 Nov 15

[10] Adzhubei IA, Schmidt S, Peshkin L, Ramensky VE, Gerasimova A, Bork P, et al. A method and server for predicting damaging missense mutations. Nat Methods 2010;7:248-9.

[11] Ng PC, Henikoff S. Predicting deleterious amino acid substitutions. Genome Res 2001;11:863-74.

[12] Tavtigian SV, Deffenbaugh AM, Yin L, Judkins T, Scholl T, Samollow PB, et al. Comprehensive statistical study of 452 BRCA1 missense substitutions with classification of eight recurrent substitutions as neutral. J Med Genet 2006;43:295-305.

[13] Choi Y, Sims GE, Murphy S, Miller JR, Chan AP. Predicting the functional effect of amino acid substitutions and indels. PLoS One 2012;7:e46688.

[14] Desmet FO, Hamroun D, Lalande M, Collod-Béroud G, Claustres M, Béroud C. Human Splicing Finder: an online bioinformatics tool to predict splicing signals. Nucleic Acids Res 2009;37:e67.

[15] 1000 Genomes Project Consortium, Abecasis GR, Auton A, Brooks LD, DePristo MA, Durbin RM, et al. An integrated map of genetic variation from 1,092 human genomes. Nature 2012; 491:56-65.

[16] Exome Aggregation Consortium (ExAC) Databases. <http://exac.broadinstitute.org>. Accessed 31 Nov 15

[17] Global MAF dbSNP Databases. <http://www.ncbi.nlm.nih.gov/snp>. Accessed 31 Nov 15.

[18] Exome Variant Server, NHLBI GO Exome Sequencing Project (ESP) Databases. <http://evs.gs.washington.edu/EVS/>. Accessed 31 Nov 15.

[19] Richards S, Aziz N, Bale S, Bick D, Das S, Gastier-Foster J, et al. Standards and guidelines for the interpretation of sequence variants: a joint consensus recommendation of the American College of Medical Genetics and Genomics and the Association for Molecular Pathology. Genet Med 2015;17:405-24.

| **Additional Table 1.** Oligonucleotides for *BRCA1* gene sequencing. F, forward; R, reverse. | | | | |
| --- | --- | --- | --- | --- |
| ***BRCA1* Exon** | **Primer** | **MgCl (mM)** | **°C annealing** | **Length (bp)** |
| 2 | F: 5’ GAAGTTGTCATTTTATAAACCTTT 3’ | 1.8 | 54 | 250 |
|  | R: 5’ TGTCTTTTCTTCCCTAGTATG 3’ |  |  |  |
| 3 | F: 5’ TCCTGACACAGCAGACATTTA 3’ | 1.2 | 56 | 340 |
|  | R: 5’ TTGGATTTTTCGTTCTCACTTA 3’ |  |  |  |
| 5 | F: 5’ GCTTGTAATTCACCTGCCAT 3’ | 1.8 | 58 | 270 |
|  | R: 5’ TTCCTACTGTGGTTGCTTCC 3’ |  |  |  |
| 6 | F: 5’ AGGTTTTCTACTGTTGCTGCAT 3’ | 1.2 | 66 | 308 |
|  | R: 5’ AAAAGGTCTTATCACCACGTCA 3’ |  |  |  |
| 7 | F: 5’ GGGTTTCTCTTGGTTTCTTTG 3’ | 1.2 | 66 | 329 |
|  | R: 5’ GGAGGACTGCTTCTAGCCTG 3’ |  |  |  |
| 8 | F: 5’ AAGCACAGAACTGGCCAACAA 3’ | 1.2 | 66 | 274 |
|  | R: 5’ CACTTCCCAAAGCTGCCTAC 3’ |  |  |  |
| 9 | F: 5’ TGCCACAGTAGATGCTCAGT 3’ | 1.2 | 64 | 292 |
|  | R: 5’ CACATACATCCCTGAACCTAAA 3’ |  |  |  |
| 10 | F: 5’ TTGGTCAGCTTTCTGTAATCG 3’ | 1.2 | 62 | 348 |
|  | R: 5’ CCATACCACGACATTTGACA 3’ |  |  |  |
| 11 A | F: 5’ TAGCCAGTTGGTTGATTTCC 3’ | 1.2 | 64 | 394 |
|  | R: 5’ CCCATCTGTTATGTTGGCTC 3’ |  |  |  |
| 11 B | F: 5’ CCATGTGGCACAAATACTCA 3’ | 1.2 | 64 | 399 |
|  | R: 5’ TGATTCAGACTCCCCATCAT 3’ |  |  |  |
| 11 C | F: 5’ GAAACTGCCATGCTCAGAGA 3’ | 1.2 | 64 | 437 |
|  | R: 5’ ATTTATTTGTGAGGGGACGC 3’ |  |  |  |
| 11 D | F: 5’ TCCCCAACTTAAGCCATGTA 3’ | 1.2 | 64 | 437 |
|  | R: 5’ AGAAGACTTCCTCCTCAGCC 3’ |  |  |  |
| 11 E | F: 5’ TTCAAAACGAAAGCTGAACC 3’ | 1.2 | 64 | 444 |
|  | R: 5’ TTGGAAGGCTAGGATTGACA 3’ |  |  |  |
| 11 F | F: 5’ GGTAAAGAACCTGCAACTGG 3’ | 1.2 | 64 | 416 |
|  | R: 5’ TCAAATGCTGCACACTGACT 3’ |  |  |  |
| 11 G | F: 5’ GAAAGGGTTTTGCAAACTGA 3’ | 1.8 | 64 | 381 |
|  | R: 5’ TTCTCTTTCTGCATTTCCTG 3’ |  |  |  |
| 11 H | F: 5’ TTTCGTTGCCTCTGAACTGA 3’ | 1.2 | 64 | 376 |
|  | R: 5’ AACCACAGTCGGGAAACA 3’ |  |  |  |
| 11 I | F: 5’ CTCAGGTTGCAAAACCCCTA 3’ | 1.2 | 64 | 507 |
|  | R: 5’ ATCACTGCAGGCTTTCCTGT 3’ |  |  |  |
| 11 J | F: 5’ ACTAATGAAGTGGGCTCCAG 3’ | 1.2 | 64 | 445 |
|  | R: 5’ CCAAATGTGTATGGGTGAAA 3’ |  |  |  |
| 11 K | F: 5’ GATGTTCCTGAGATGCCTTTG 3’ | 1.2 | 64 | 401 |
|  | R: 5’ TGATGACCTGTTAGATGATGGTG 3’ |  |  |  |
| 11 L | F: 5’ ACCGTTGCTACCGAGTGTCT 3’ | 1.2 | 64 | 438 |
|  | R: 5’ GTGCTCCCCAAAAGCATAAA 3’ |  |  |  |
| 12 | F: 5’ GTCCTGCCAATGAGAAGAAAAAG 3’ | 1.2 | 64 | 267 |
|  | R: 5’ TGTCAGCAAACCTAAGAATGT 3’ |  |  |  |
| 13 | F: 5’ AATGGAAAGCTTCTCAAAGTA 3’ | 1.2 | 58 | 322 |
|  | R: 5’ ATGTTGGAGCTAGGTCCTTAC 3’ |  |  |  |
| 14 | F: 5’ TGTGTATCATAGATTGATGCTTTTG 3’ | 1.2 | 60 | 360 |
|  | R: 5’ GCAATAAAAGTGTATAAATGCCTGT 3’ |  |  |  |
| 15 | F: 5’ TTGCCAGTCATTTCTGATCT 3’ | 1.2 | 60 | 484 |
|  | R: 5’ AAACCTTGATTAACACTTGAGC 3’ |  |  |  |
| 16 | F: 5’ AATTCTTAACAGAGACCAGAAC 3’ | 1.2 | 60 | 452 |
|  | R: 5’ AAAACTCTTTCCAGAATGTTGT 3’ |  |  |  |
| 17 | F: 5’ GTGTAGAACGTGCAGGATTG 3’ | 1.2 | 56 | 265 |
|  | R: 5’ TCGCCTCATGTGGTTTTA 3’ |  |  |  |
| 18 | F: 5’ GGCTCTTTAGCTTCTTAGGAC 3’ | 1.2 | 62 | 354 |
|  | R: 5’ GAGACCCATTTTCCCAGCATC 3’ |  |  |  |
| 19 | F: 5’ CTGTCATTCTTCCTGTGCTC 3’ | 1.2 | 62 | 250 |
|  | R: 5’ CATTGTTAAGGAAAGTGGTGC 3’ |  |  |  |
| 20 | F: 5’ TGCTAGGATTACAGGGGTGAG 3’ | 1.2 | 62 | 322 |
|  | R: 5’ TTTATGTGGTGGGGATGGAAG 3’ |  |  |  |
| 21 | F: 5’ CAGGTGGTGAACAGAAGAAA 3’ | 1.2 | 58 | 300 |
|  | R: 5’ ACATTTCAGCAATCTGAGGA 3’ |  |  |  |
| 22 | F: 5’ CATCCGGAGAGTGTAGGGTA 3’ | 1.2 | 58 | 240 |
|  | R: 5’ CATCCATAGGGACTGACAGG 3’ |  |  |  |
| 23 | F: 5’ CCCTGTCTCAAAAACAAACA 3’ | 1.8 | 60 | 235 |
|  | R: 5’ CAAGCACCAGGTAATGAGTG 3’ |  |  |  |
| 24 | F: 5’ TGGAGTCGATTGATTAGAGC 3’ | 1.2 | 58 | 301 |
|  | R: 5’ AGCCAGGACAGTAGAAGGAC 3’ |  |  |  |

| **Additional Table2 .** Oligonucleotides for *BRCA2* gene sequencing. F, forward; R, reverse. | | | | |
| --- | --- | --- | --- | --- |
| ***BRCA2* Exon** | **Primer** | **MgCl (mM)** | **°C annealing** | **Length (bp)** |
| 2 | F: 5’ CCAGGAGATGGGACTGAATTAG 3’ | 1.2 | 64 | 311 |
|  | R: 5’ CTGTGACGTACTGGGTTTTTAGC 3’ |  |  |  |
| 3 | F: 5’ TTCCTTATGATCTTTAACTGTTCTG 3’ | 1.8 | 64 | 406 |
|  | R: 5’ GCTAAGATTTTAACACAGGTTTGC 3’ |  |  |  |
| 4 | F: 5’ AGAATGCAAATTTATAATCCAGAGTA 3’ | 1.5 | 60 | 249 |
|  | R: 5’ AATCAGATTCATCTTTATAGAACAAA 3’ |  |  |  |
| 5+6 | F: 5’ TTCCAACAATTTATATGAATGAGAATC 3’ | 1.8 | 62 | 362 |
|  | R: 5’ CTCAGGGCAAAGGTATAACGC 3’ |  |  |  |
| 7 | F: 5’ CCTTAATGATCAGGGCATTTC 3’ | 1.2 | 64 | 214 |
|  | R: 5’ CAACCTCATCTGCTCTTTCTTG 3’ |  |  |  |
| 8 | F: 5’ GTAGATGTGCTTTTTGATGTCTGAC 3’ | 1.2 | 64 | 315 |
|  | R: 5’ GAGAGACAGCAGAGTTTCACAGG 3’ |  |  |  |
| 9 | F: 5’ CAGATAACTGAAATCACCAAAAGTG 3’ | 1.2 | 62 | 262 |
|  | R: 5’ ACAACAACAAAAAAACCTGTAGTTC 3’ |  |  |  |
| 10A | F: 5’ TATAAAATATTAATGTGCTTCTGTT 3’ | 1.2 | 54 | 374 |
|  | R: 5’ AAAGGGCTTCTGATTTGCTAC 3’ |  |  |  |
| 10B | F: 5’ ATCTGAAGTGGAACCAAATGATAC 3’ | 1.2 | 64 | 280 |
|  | R: 5’ ACGTGGCAAAGAATTCTCTGAAGTAA 3’ |  |  |  |
| 10C | F: 5’ TTTCAGAAAAAGACCTATTAGACA 3’ | 1.5 | 60 | 242 |
|  | R: 5’ CTTTTTGATACCCTGAAATGAAGAAG 3’ |  |  |  |
| 10D | F: 5’ TAAAGCAGGCAATATCTGGAACTTCT 3’ | 1.2 | 60 | 295 |
|  | R: 5’ GTGGATATTAAACCTGCATTCTTCAA 3’ |  |  |  |
| 10E | F: 5’ TTTAATTGATAATGGAAGCTGG 3’ | 1.2 | 58 | 268 |
|  | R: 5’ TTACAAAAAAAAAAAGACAGAGGT 3’ |  |  |  |
| 11A | F: 5’ TTGTCAGATTTAACTTTTTTGGAAG 3’ | 1.2 | 63-56 | 342 |
|  | R: 5’ CAACTGGGACACTTTCTTTCAG 3’ |  |  |  |
| 11B | F: 5’ GCTCAAGAAGCATGTCATGG 3’ | 1.2 | 63-56 | 394 |
|  | R: 5’ TATGAAAACCCAACAGAGTAGGT 3’ |  |  |  |
| 11C | F: 5’ GAAAGAAAGTGTCCCAGTTG 3’ | 1.2 | 63-56 | 360 |
|  | R: 5’ ACCACAGTCTCAATAGAAACAAGG 3’ |  |  |  |
| 11D | F: 5’ TAGTCACAAGTTCCTCAACGA 3’ | 1.2 | 63-56 | 709 |
|  | R: 5’ TGAGACCATTGAGATCACAGC 3’ |  |  |  |
| 11E | F: 5’ TGATTGATGGTACTTTAATTTTGTCAC 3’ | 1.2 | 63-56 | 338 |
|  | R: 5’ AGCCAAGACCTCTTCTTTTATATCTG 3’ |  |  |  |
| 11F | F: 5’ AAGCTGATTCTCTGTCATGCCTG 3’ | 1.8 | 63-56 | 456 |
|  | R: 5’ GATTTGTGTTTTGGTTGAATTGTACC 3’ |  |  |  |
| 11G | F: 5’ AAAATACATGAGAGTAGCATCACC 3’ | 1.8 | 63-56 | 330 |
|  | R: 5’ AAATCTTTTTTAATTGACACTTGG 3’ |  |  |  |
| 11H | F: 5’ CGAACCCATTTTCAAGAACTCTACCA 3’ | 1.2 | 63-56 | 215 |
|  | R: 5’ TGTAATCATTATTTTTTTCTGG 3’ |  |  |  |
| 11I | F: 5’ TTGGTTTATGTTCTTGCAGAGGAG 3’ | 1.2 | 63-56 | 487 |
|  | R: 5’ CCTTTTGGCTAGGTGTTAAATTATGG 3’ |  |  |  |
| 11J | F: 5’ TGGCATTAGATAATCAAAAGAAACTG 3’ | 1.2 | 63-56 | 500 |
|  | R: 5’ CCTAAACCCCACTTCATTTTCATC 3’ |  |  |  |
| 11 K | F: 5’ TGAATCACTGCCATCAAATTCTAA 3’ | 1.2 | 63-56 | 465 |
|  | R: 5’ GAAATTAAACGGAAGTTTGCTGG 3’ |  |  |  |
| 11L | F: 5’ AATGACTACTGGCACTTTTGTTG 3’ | 1.2 | 68-61 | 401 |
|  | R: 5’ CACTTGCAGTCTGAAAAAATGTATC 3’ |  |  |  |
| 11M | F: 5’ GCCAGTATTGAAGAATGTTGAAGATC 3’ | 1.2 | 68-61 | 443 |
|  | R: 5’ AAACCTTATGTGAATGCGTGCTAC 3’ |  |  |  |
| 11N | F: 5’ AACGAAAATTATGGCAGGTTGTTAC 3’ | 1.2 | 68-61 | 436 |
|  | R: 5’ GCTTTCCACTTGCTGTACTAAATCC 3’ |  |  |  |
| 11O | F: 5’ CCAGCTCACAAGAGAAGAAAATACTG 3’ | 1.2 | 68-61 | 503 |
|  | R: 5’ TTACGTTTTTAGGTGAAGCCTGTTC 3’ |  |  |  |
| 11P | F: 5’ AAACCCAGAGCACTGTGTAAACTC 3’ | 1.5 | 68-61 | 487 |
|  | R: 5’ TCTCCTCTTCTTTTTCCAATTCTTG 3’ |  |  |  |
| 11Q | F: 5’ TACAGATTCTAAACTGCCAAGTCATG 3’ | 1.5 | 68-61 | 265 |
|  | R: 5’ TAACCATACTCCCCCAAACTGAC 3’ |  |  |  |
| 12 | F: 5’ AATTGACATTGAAGACTGACTTTACTC 3’ | 1.5 | 64 | 370 |
|  | R: 5’ AGCACTTTGGAGAGGCAGG 3’ |  |  |  |
| 13 | F: 5’ GCATCCGTTACATTCACTGAAA 3’ | 1.5 | 64 | 310 |
|  | R: 5’ ACGGGAAGTGTTAACTTCTTAACG 3’ |  |  |  |
| 14A | F: 5’ ACCATGTAGCAAATGAGGGTCT 3’ | 1.5 | 62 | 391 |
|  | R: 5’ GCTTTTGTCTGTTTTCCTCCAA 3’ |  |  |  |
| 14B | F: 5’ CACAGAGTTGAACAGTGTGTTAGG 3’ | 1.5 | 62 | 297 |
|  | R: 5’ GGGCTTTAAAATTACCACCACC 3’ |  |  |  |
| 15 | F: 5’ GGCCAGGGGTTGTGCTTTTT 3’ | 1.2 | 60 | 369 |
|  | R: 5’ ATTTCATTCATCCATTCCTGC3’ |  |  |  |
| 16 | F: 5’ TTTGGTAAATTCAGTTTTGGTTTG 3’ | 1.2 | 60 | 396 |
|  | R: 5’ AGCCAACTTTTTAGTTCGAGAG 3’ |  |  |  |
| 17 | F: 5’ CAGAGAATAGTTGTAGTTGTTGAA 3’ | 1.5 | 62 | 306 |
|  | R: 5’ AGAAACCTTAACCCATACTGC 3’ |  |  |  |
| 18A | F: 5’ TCAGTTTTTATTCTCAGTTATTCAGTG 3’ | 1.8 | 62 | 298 |
|  | R: 5’ GCATACCACCCATCTGTAAGTTC 3’ |  |  |  |
| 18B | F: 5’ TGTTTCTGACATAATTTCATTGAGC 3’ | 1.8 | 62 | 420 |
|  | R: 5’ AAACTTTAACTGTCTGAAGAATATGC 3’ |  |  |  |
| 19 | F: 5’ CTTATTTACTGTCTTACTAATCTTCCT 3’ | 1.8 | 64 | 389 |
|  | R: 5’ GACCGAAACTCCATCTCAAAC 3’ |  |  |  |
| 20 | F: 5’ GGTGATCCACTAATCTCAGCCTC 3’ | 1.8 | 66 | 451 |
|  | R: 5’ GTCCCTTGTTGCTATTCTTTGTCT 3’ |  |  |  |
| 21 | F: 5’ GGGTGTTTTATGCTTGGTTCT 3’ | 1.5 | 58 | 303 |
|  | R: 5’ CATTTCAACATACTCCTTCCTG 3’ |  |  |  |
| 22 | F: 5’ AACCACACCCTTAAGATGAGC 3’ | 1.5 | 62 | 455 |
|  | R: 5’ GGGCATTAGTAGTGGATTTTGC 3’ |  |  |  |
| 23 | F: 5’ ACTTCTTCCATTGCATCTTTCTCA 3’ | 1.5 | 58 | 290 |
|  | R: 5’ AAAACAAAACAAAAATTCAACATA 3’ |  |  |  |
| 24 | F: 5’ GCAGCGACAAAAAAAACTCA 3’ | 1.5 | 64 | 365 |
|  | R: 5’ ATTTGCCAACTGGTAGCTCC 3’ |  |  |  |
| 25 | F: 5’ GCTTTCGCCAAATTCAGCTA 3’ | 1.5 | 58 | 427 |
|  | R: 5’ TACCAAAATGTGTGGTGATGC 3’ |  |  |  |
| 26 | F: 5’ GTCCCAAACTTTTCATTTCTGC 3’ | 1.8 | 64 | 379 |
|  | R: 5’ GGAGCCACATAACAACCACA 3’ |  |  |  |
| 27A | F: 5’ CTGTGTGTAATATTTGCGTGCT 3’ | 1.2 | 62 | 495 |
|  | R: 5’ GCAAGTTCTTCGTCAGCTATTG 3’ |  |  |  |
| 27B | F: 5’ GAATTCTCCTCAGATGACTCCA 3’ | 1.8 | 62 | 417 |
|  | R: 5’ TCTTTTCTCATTGTGCAACA 3’ |  |  |  |
|  | | | | |
